# Supplementary material for: COVID-19 Vaccines Safety Tracking (CoVaST): Protocol of a Multi-Center Prospective Cohort Study for Active Surveillance of COVID-19 Vaccines’ Side Effects
Source: Int J Environ Res Public Health. 2021 Jul 25;18(15):7859. doi: 10.3390/ijerph18157859 (PMC8345554; doi:10.3390/ijerph18157859)
Supplement: Supplementary file 1 [file ijerph-18-07859-s001.zip › ijerph-1257063-SI.pdf]

Table S1: CoVaST Instrument.

**1. Demographic Data**	
Gender	o Female<br>o Male<br>o Prefer not to say
Age	o Dropdown menu with numbers (18-99)
Profession	o Physician (M.D.)<br>o Dentist (D.D.S)<br>o Nurse (R.N.)<br>o Pharmacist (PharmD)<br>o Physiotherapist<br>o Other (please specify)
Region	o All the regions of the participating country should be enlisted here. For EU members, NUTS-3 level categorization should be followed.
Weight	kg
Height	cm
**2. Medical Anamnesis**	
Do you have any chronic disease?	o Yes<br>o No
If "Yes", please specify all chronic diseases you suffer from currently	o Allergy<br>o Asthma<br>o Blood Disease<br>o Bowel Disease<br>o Cancer<br>o Cardiac Disease<br>o Chronic Hypertension<br>o COPD<br>o Diabetes Mellitus – I<br>o Diabetes Mellitus – II<br>o Hepatologic Disease<br>o Psychological Distress<br>o Neurologic Disease<br>o Ophthalmologic Disease<br>o Renal Disease<br>o Rheumatoid Arthritis

	o Thyroid Disease<br>o Other, please specify
Do you take any medication currently?	o Yes<br>o No
If "Yes", please specify the category of the drug	o Anti-asthma<br>o Antibiotics<br>o Anticoagulant<br>o Antidepressant<br>o Antidiabetic<br>o Antiepileptic<br>o Antihistamine<br>o Antihypertensive<br>o Anti-Reflux<br>o Anti-venous Insufficiency<br>o Immunosuppressive<br>o Cholesterol-lowering<br>o Common Analgesic<br>o Contraceptive<br>o Corticosteroid<br>o NSAID<br>o Opioid Analgesic<br>o Thyroid Hormones<br>o Other, please specify (the generic name or the market name of the drug)
Do you smoke cigarettes?	o Yes<br>o No
If "Yes", how many cigarettes do you smoke per day?	o Dropdown menu with numbers (0-99)
Do you drink alcohol?	o Yes<br>o No
If "Yes", how many glasses of (0.5 l) beer per week?<br>If "Yes", how many glasses of (0.2 l) Wine per week?<br>If "Yes", how many glasses of (0.04 l) Spirit per week?	o Dropdown menu with numbers (0-99)<br>o Dropdown menu with numbers (0-99)<br>o Dropdown menu with numbers (0-99)
**3. COVID-19-related Anamnesis**	
Vaccine Type	o Oxford–AstraZeneca COVID-19 Vaccine<br>o Pfizer-BioNTech COVID-19 Vaccine<br>o Moderna COVID-19 Vaccine<br>o Janssen Vaccine<br>o Sputnik V Vaccine

	o Covaxin Vaccine<br>o Other, please specify
Vaccination Date (first dose)	o Please select the date from the (Calendar)
Have you taken the second dose?	o Yes<br>o No
Vaccination Date (second dose)	o Please select the date from the (Calendar)
Have you ever been diagnosed with COVID-19?	o Yes<br>o No
If "Yes", when were you diagnosed?	o Before vaccination<br>o Between 1st and 2nd dose of vaccine<br>o After vaccination
Please specify the date when you were diagnosed	o Please select the date from the (Calendar)
How do you describe the severity of your COVID-19 infection?	o **Mild** (no symptoms, or mild upper respiratory tract symptoms, or cough, new myalgia, or asthenia without new shortness of breath or a reduction in oxygen saturation)<br>o **Moderate** (prostration, severe asthenia, fever > 38 ℃ or persistent cough clinical or radiological signs of lung involvement no clinical or laboratory indicators of clinical severity or respiratory impairment)<br>o **Severe** (respiratory rate ≥ 30 breaths/min, or oxygen saturation ≤ 92% at a rest state, or arterial partial pressure of oxygen (PaO2)/ inspired oxygen fraction (FiO2) ≤ 300)<br>o **Critical** (Respiratory failure Occurrence of severe respiratory failure (PaO2/FiO2 < 200), respiratory distress or acute respiratory distress syndrome (ARDS). This includes patients deteriorating despite advanced forms of respiratory support (non-invasive ventilation (NIV), high-flow nasal oxygen (HFNO)) OR patients requiring mechanical ventilation. OR other signs of significant deterioration hypotension or shock impairment of consciousness other organ failure)

What were the symptoms you have experienced during the COVID-19 infection?	o Fever or chills<br>o Cough<br>o Shortness of breath or difficulty breathing<br>o Fatigue<br>o Muscle or body aches<br>o Headache<br>o New loss of taste or smell<br>o Sore throat<br>o Congestion or runny nose<br>o Nausea or vomiting<br>o Diarrhea<br>o Other (please specify)
For how many days did you experience the COVID-19 symptoms?	o Dropdown menu with numbers (0-99)
**4. Vaccine Side Effects**	
Within four weeks of receiving the vaccine, have you suffered from any of the following local side effects?	• Injection site pain<br>• Injection site swelling<br>• Injection site redness<br>• Other, please specify
When did the local side effects emerge?	o After the first dose only<br>o After the second dose only<br>o After both doses
If you chose any of the previous side effects, please indicate their duration	o 1 day<br>o 2 days<br>o 3 days<br>o 5 days<br>o 1 week<br>o 2 weeks<br>o 3 weeks<br>o 4 weeks<br>o > 1 month
Within four weeks of receiving the vaccine, have you suffered from any of the following side effects?	o None<br>o Fatigue<br>o Headache<br>o Muscle Pain<br>o Joint Pain<br>o Fever<br>o Chills<br>o Nausea<br>o Diarrhoea<br>o Shortness of breath<br>o Anaphylaxis

	o Swollen lymph nodes<br>o Mouth tingling<br>o Loss of taste<br>o Change of taste<br>o Halitosis (Oral malodour)<br>o Oral ulcers / blisters / vesicles<br>o Bleeding gingiva<br>o Skin rash<br>o Other, please specify
When did the systemic side effects emerge?	o After the first dose only<br>o After the second dose only<br>o After both doses
If you chose any of the previous side effects, please indicate their duration	o 1 day<br>o 2 days<br>o 3 days<br>o 5 days<br>o 1 week<br>o 2 weeks<br>o 3 weeks<br>o 4 weeks<br>o > 1 month
Have you taken any medications to relieve your side effects?	o Yes<br>o No
If "Yes", please specify what drug you have used. Use either generic name or market name	
Do you agree to participate in the longitudinal study evaluating the safety of the vaccines from a long term perspective?	o Yes<br>No
If yes, give us please your contact e-mail address. Your e-mail address will be automatically stored and removed from the survey so the data you have shared will remain anonymous.	
